# NF-κB and CREB Are Required for Angiotensin II Type 1 Receptor Upregulation in Neurons

**DOI:** 10.1371/journal.pone.0078695

**Published:** 2013-11-11

**Authors:** Karla K. V. Haack, Amit K. Mitra, Irving H. Zucker

**Affiliations:** Department of Cellular and Integrative Physiology, University of Nebraska Medical Center, Omaha, Nebraska, United States of America; University of São Paulo, Brazil

## Abstract

Nuclear factor kappa B (NF-κB) and the Ets like gene-1 (Elk-1) are two transcription factors that have been previously established to contribute to the Angiotensin II mediated upregulation of Angiotensin II type 1 receptor (AT1R) in neurons. The cAMP response element binding protein (CREB) is another transcription factor that has also been implicated in AT1R gene transcription. The goal of the current study was to determine if NF-κB and CREB association was required for AT1R upregulation. We hypothesized that the transcription of the AT1R gene occurs via an orchestration of transcription factor interactions including NF-κB, CREB, and Elk-1. The synergistic role of CREB and NFκB in promoting AT1R gene expression was determined using siRNA-mediated silencing of CREB. Electrophorectic Mobility Shift Assay studies employing CREB and NF-κB demonstrated increased protein – DNA binding as a result of Ang II stimulation which was blunted by siRNA silencing of CREB. Upstream inhibition of p38 mitogen activated protein kinase (p38 MAPK) with SB203580 or inhibition of the calmodulin kinase (CAMK) pathway using KN-62 blunted changes in CREB and NF-κB expression. These findings suggest that Ang II may activate multiple signaling pathways involving p38 MAPK leading to the activation of NF-κB and CREB, which feed back to upregulate the AT1R gene. This study provides insight into the molecular mechanisms involving multiple transcription factor activation in a coordinated fashion which may be partially responsible for sympathoexcitation in clinical conditions associated with increased activation of the renin angiotensin system.

## Introduction

It is well known that the renin-angiotensin system (RAS) plays a central role in mediating the increased sympathetic outflow observed in cardiovascular diseases such as hypertension and heart failure [1]. High levels of circulating Angiotensin II (Ang II) together with increased upregulation of the Angiotensin II type 1 receptor (AT_1_R) in cardiovascular regulatory regions of the brain such as the rostral ventrolateral medulla (RVLM) contribute to this sympathoexcitation [2], [3]. Ang II is known to activate numerous nuclear transcription factors such as nuclear factor kappa B (NF-κB) [4], activator protein 1 (AP-1) [5], and Ets-like protein 1 (Elk-1) [6]. We have shown previously that NF-κB and Elk-1 are two of the key players required for the transcription of AT_1_R in a CATH.a neuronal cell line [7]. *In vivo* studies from our laboratory have also shown that in a rat model of chronic heart failure, central AT_1_R upregulation was dependent on the transcription factors c-Fos and c-Jun, two components of AP-1 [8]. Taken together, these data suggest a cascade of transcription factors that constitute a positive feedback system for the upregulation of AT_1_R.

Angiotensin II stimulation has also been shown to phosphorylate the cAMP-response element binding protein (CREB) via activation of the p38 MAPK pathway in hypertension [9], [10], [11], [12]. Other signaling cascades that lead to the phosphorylation of CREB include the calcium/Calmodulin kinase (CAMK) pathway [13], extracellular signal regulated protein kinase (ERK) activation [14], protein kinase A [15], and the p38 mitogen activated protein kinase (p38MAPK) pathway [16]. Phosphorylation at Serine 133 results in recruitment of a transcriptional co-activator, CREB-binding protein (CBP) which is essential for transcriptional activation [17]. Studies in neurons have demonstrated the presence of CREB in a functional and regulatory role especially in the mitochondria [18], [19].

Increasing evidence suggests that CREB and NF-κB work synergistically to recruit CBP, thereby leading to transcription of multiple genes [20]. The CREB promoter contains binding sites for CREB in close proximity to NF-κB motifs and NF-κB has been shown to interact with other members of the CREB family including c-Fos and c-Jun [21], [22]. Co-immunoprecipitation studies have also demonstrated that NF-κB does not directly interact with CREB; instead, CBP serves as a scaffold in NF-κB and CREB in stimulating transcription from the NF-κB promoter [23].

In the current study we tested the hypothesis that in catecholaminergic CATH.a neurons, the upregulation of AT_1_R gene transcription in response to Ang II is initiated by CREB/NF-κB activation and propagated by downstream transcription factors Elk-1 and c-Fos (a component of AP-1 known to contribute to AT_1_R upregulation).

## Materials and Methods

### Cell Culture

The locus coeruleus-like cell line CATH.a neurons (American Type Culture Collection, Manassas, VA) were cultured as described previously [7]. Briefly, the cells were grown in RPMI 1640 media containing fetal bovine serum (4%), horse serum (8%) and penicillin (100 IU/l) at 37°C and 5% CO_2._ Cells were seeded on 100 mm petri dishes (1×10^7^ cells) or 6-well plates (1.5×10^6^/well). Experiments were performed at 70%–80% confluence and the cells were differentiated in serum-free media for 48–72 hours. Cells were treated with Ang II (Sigma-Aldrich, St. Louis, MO) over specified time periods following which they were harvested and used for further investigation. For each time point beyond 8 hrs, cells were spiked with an additional dose of Ang II at 8 hours and at 16 hours (if applicable). The following inhibitors were used to determine the specific upstream molecular pathways: SB203580 (p38MAPK), KN-62 (Ca^2+^/calmodulin-dependent protein kinase II), losartan (AT_1_R) (Sigma-Aldrich), PD98059 (ERK) (Cell Signaling Technology, Danvers, MA).

### Western Blot Analysis

Cells were washed in PBS and lysed with RIPA buffer containing 1% (v/v) Nonidet P40, 0.5% SDS and protease inhibitor cocktail containing 10 µg/ml PMSF, 2 µg/ml leupeptin, 2 µg/ml pepstatin A and 2 µg/ml aprotinin. Cells were disrupted by intermittent sonication. After centrifugation, the amount of protein in the supernatant was measured by a bicinchocinic acid (BCA) protein kit (Pierce, Rockford, IL) using BSA as a standard. Cell lysates were then subjected to SDS/PAGE followed by Western blotting as described previously [24]. Primary antibodies used were p65, CREB, CBP, GAPDH (Santa Cruz Biotechnology Inc., Santa Cruz, CA) and p-CREB (Cell Signaling Technology Inc., Danvers, MA) at a dilution of 1∶500–1∶1000. Antigen–antibody complexes were detected by HRP (horseradish peroxidase)-linked appropriate secondary antibodies with a Supersignal West Pico Chemiluminescent Detection kit (Pierce, Rockford, IL) and images were digitized and analyzed by UVP Bioimaging System (UVP, Upland, CA).

### RT-PCR

Following specific treatment and time course, total RNA was isolated from the neurons using the RNeasy (Qiagen, Inc. Valencia, CA) RNA isolation kit as per manufacturer’s guidelines. RT-PCR was carried out using using 1 ug of total RNA. The purity of the RNA used had a 260/280 ratio of 1.8–2.0. PCR was carried out in a thermocycler (PTC-100, BioRad Hercules, CA,) with the following oligonucleotide primers:

CREB: Forward 3′ TACAGGGCCTGCAGACATTAACCA 5′.

Reverse 5′ ATTCTCTTGCTGCCTCCCTGTTCT 3′.

GAPDH: Forward 3′ TGATGCTGGTGCTGAGTATGTCGT 5′.

Reverse 5′ TTGTCATTGAGAGCAATGCCAGCC 3′.

cDNA synthesis was performed using iSCRIPT (BioRad, Hercules, CA) kit using the following parameters: 5 min at 25°C, 30 min at 42°C, 5 min at 85°C. PCR Mastermix (Promega, Madison, WI) was used as per the manufacturers’ guidelines using the specific primer pairs shown above. The reaction was carried out using the following parameters: 95°C for 5 minutes, 95°C for 30 seconds, 65°C for 30 seconds, 72°C for 1 minute, steps 2, 3 and 4 were repeated for 25–30 cycles followed by 10 minute incubation at 72°C and held at 4°C till gel electrophoresis on an agarose gel (1.5%) and visualized by ethidium bromide staining. The bands were analyzed using UVP Bio-imaging Systems – Biochemi 500(Upland, CA). GAPDH was used for normalization.

### Cytoplasmic-nuclear Fractionation

This was done using NE-PER kit (Pierce, Rockford, IL) as per the manufacturer’s instruction. Briefly the cells (10^8^/ ml) were resuspended in CER I lysis buffer and vortexed for 15 seconds followed by incubation on ice. CER II buffer was added (5.5% volume) and further vortexed to ensure complete mixing and lysis of cells. The cell suspension was centrifuged and the supernatant yielded the cytoplasmic fraction. To the remaining nuclear pellet NER I buffer was added (50% OF CER I volume) and further vortexed for nuclear membrane lysis. The suspension was centrifuged and the supernatant collected as the nuclear fraction. Total protein in both fractions was determined by BCA assay, and equivalent proteins were loaded. GAPDH was used as a control for normalizing total protein (data not shown).

### Dual Luciferase Reporter Assay

Cignal Reporter assay (SABiosciences, Frederick, MD) was performed as per the manufacturer’s instructions. Briefly, one day before transfection, the cells were plated in 24-well plates in 500 ul of growth medium without antibiotics, so they reached 80–90% confluence at the time of transfection. On the day of transfection, transcription factor responsive reporters for NF-κB and CREB, negative and positive control constructs were diluted in Opti-MEM (Invitrogen, Grand Island, NY) medium without serum and antibiotics. The diluted reporter constructs and the Lipofectamine were then mixed together and allowed to stand at room temperature for 20 minutes for the formation of the DNA-Lipofectamine complex and appropriate volumes were added to each well. Positive and negative controls were run on a separate plate. The cells were then incubated at 37°C for 24 hours, following which they were subjected to experimental treatments and the cells were trypsinized and harvested at specific time points. Luciferase activity was quantified using the Dual-Luciferase Assay System (Promega, Madison, WI) and TD-20/20 luminometer (Turner Biosystems, Sunnyvale, CA). The Firefly/Renilla activity ratio generated from the transcription factor-responsive reporter transfections were divided by the Firefly/Renilla activity ratio generated from the negative control transfections to obtain the relative luciferase units. Three independent transfections were carried out in triplicate for each of the conditions tested with each reporter assay.

### Electrophoretic Mobility Shift Assay (EMSA)

Assays were performed using the EMSA gel shift kit (Panomics, Fremont, CA) according to the manufacturer’s instructions. In brief, the biotin labeled EMSA probes for CREB and NF-κB binding assay was purchased from Panomics. The probe sequences were as follows:

CREB 5′ AGAGATTGCCTGACGTCAGAGAGCTAG 3′.

NF-κB 5′ AGTTGAGGGGACTTTCCCAGGC 3′.

The DNA-protein binding reaction was performed in supplemented binding buffer containing 1 µl of probe, 5 µg of nuclear extracts, and 1 µl poly(dI-dC) at 15°C for 30 min. For competition studies, the nuclear extracts were incubated with 1- or 10-fold molar excess of unlabelled competitor oligonucleotides. All the DNA-protein complexes were analyzed on native 6% (w/v) polyacrylamide gels in 0.5× TBE buffer (22.5 mM Tris-borate, 5 mM EDTA) at 120V for approximately 1 hour or until the dye front reached the bottom of the gel depending upon the fragment size. The gels were then transferred onto nylon membranes and fixed in an oven at 80°C for 1 hour and detected using streptavidin-HRP with a chemiluminescent substrate, and visualized by UVP Bio-imaging System. For supershift assays, a reaction mixture was prepared by adding 1–2 µl of appropriate antibody per 20 µl of reaction volume. The reaction mixture was incubated at 4°C overnight.

### siRNA Mediated Gene Silencing *in-vitro*

Gene silencing was used to inhibit the expression of CREB in CATH.a neurons. The ON-TARGET plus™ (Thermo Scientific) siRNA was used:

Anti-CREB sequence - 5′- CCCTTCCCACCTTCCCTACA -3′.

The protocol was as per manufacturer’s instructions. The siRNA was used at a final concentration of 0.1–0.5 µM. Transfection was done using siPORT amine (Ambion, Austin, Tx) diluted in Opti-MEM 1 serum-free medium. The controls were treated with vehicle without the siRNA oligos. The culture plates were assayed for target gene activity 24–48 hours after transfection by RT-PCR and Western blotting.

### Co-immunoprecipitation (co-IP)

100 µg of CATH.a total protein lysate was tumbled with Roche Protein G beads (Roche, Indianapolis, IN) and pre-cleared for three hours. After centrifugation (20 seconds, 12,000× g), the pre-cleared lysate was allowed to tumble with anti-CBP, anti-CREB or anti-p65 NF-κB antibody (Santa Cruz Biotechnology, Santa Cruz, CA) for 1 hr prior to the addition of Protein G beads. The conjugated beads and lysate were tumbled overnight at 4°C. The beads were washed with two separate washing buffers (Buffer 1∶50 mM Tris-HCl pH 7.5, 150 mM NaCl, 1% Tween−20, 0.05% Na Deoxycholate; Buffer 2∶50 mM Tris-HCl pH 7.5, 75 mM NaCl, 0.1% Tween-20) with protease inhibitor cocktail added on the day of experimentation. Protein was eluted from the beads using 50 µL of 2X 4% SDS sample buffer with β-mercaptoethanol (2.5% final concentration) added fresh and heated to 100°C prior to western blotting.

### Statistical Analysis

The data are expressed as mean ± standard error of mean (SEM) and analyzed using one way ANOVA with Tukey’s post-test analysis for comparison of intra as well as inter-group variance. Statistical significance was assumed when p<0.05.

## Results

### Phosphorylation of CREB at Ser133 by Ang II

**Figure 1:**
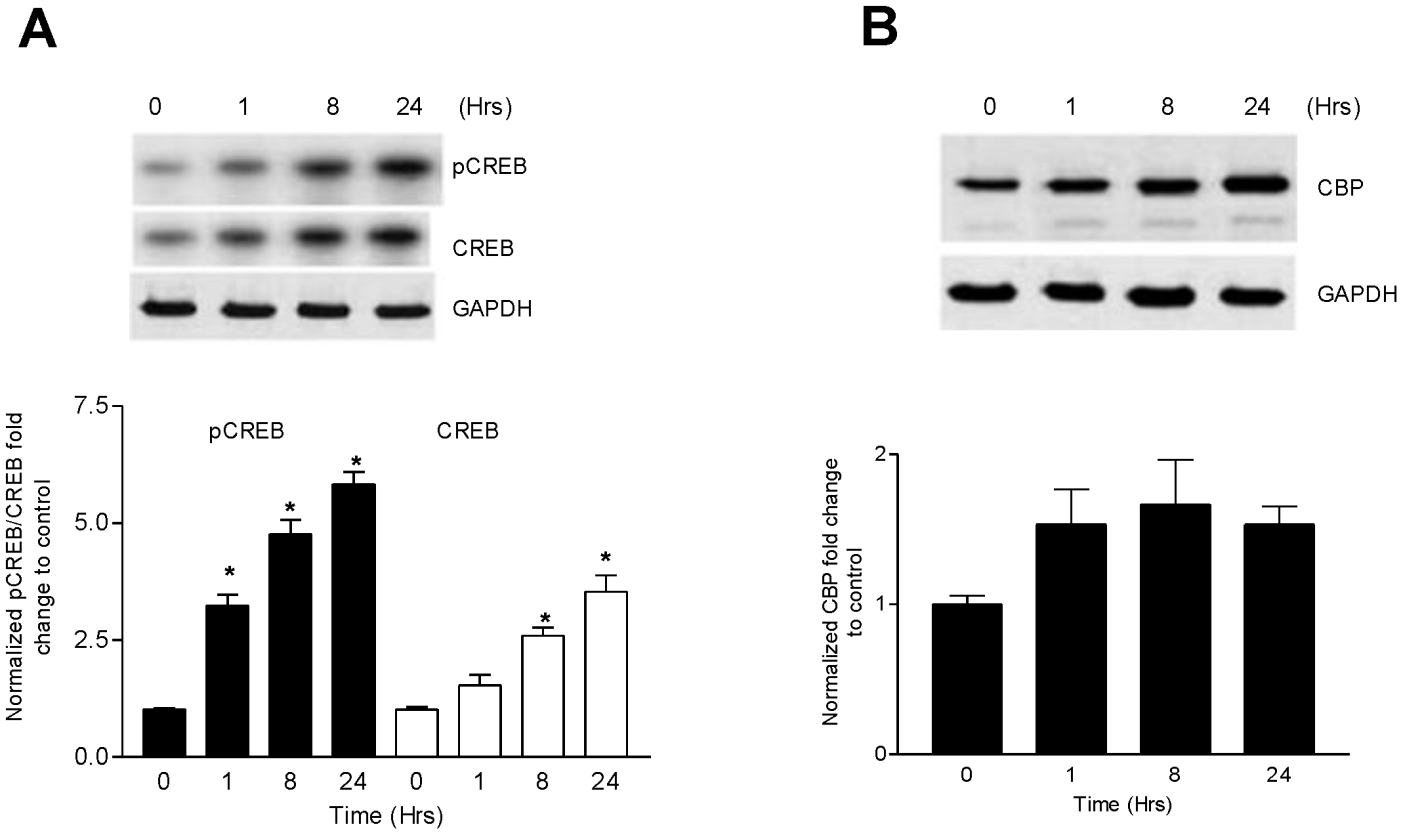
CREB is phosphorylated at Ser 133 by Angiotensin II. (A) CATH.a cells were stimulated with Ang II (100 nM) for specified time periods as indicated and immunoblotted for total and phosphorylated CREB protein using specific antibodies for total CREB and Ser 133 phosphorylation. (B) Effect of Ang II stimulation on CBP protein kinetics in CATH.a cells. The cells were stimulated with Ang II over a specified time course and the CBP concentration in the cell lysates was determined by western blot. Representative blots shown. Values are expressed as mean ± SEM. *p<0.05 vs control. n = 3–4.

To determine whether Ang II stimulation causes the phosphorylation of CREB at Ser 133 (a requirement for its activation), CATH.a cells were stimulated with Ang II (100 nM) for 1, 8, and 24 hours and the cell extracts were immunoblotted with an antibody that recognizes p-CREB at Ser133. These time points were chosen because we have shown previously that NF-κB was increased at these time points with a bi-phasic peak at 1 and 8 hours [7]. Ang II significantly increased p-CREB levels within 1 hour and sustained this increase for up to 24 hours (Fig. 1A). Total CREB was also significantly increased with 8 and 24 hour Ang II stimulation (Fig. 1A, white bars). We next asked if the increase in total and p-CREB was concomitant with CREB activation (i.e. by CBP) at these time points. Immunoblot analysis of CBP indicated no significant increase in response to Ang II (Fig. 1B).

### Multiple Kinase Pathways Mediate Ang II-induced Phosphorylation of CREB

**Figure 2:**
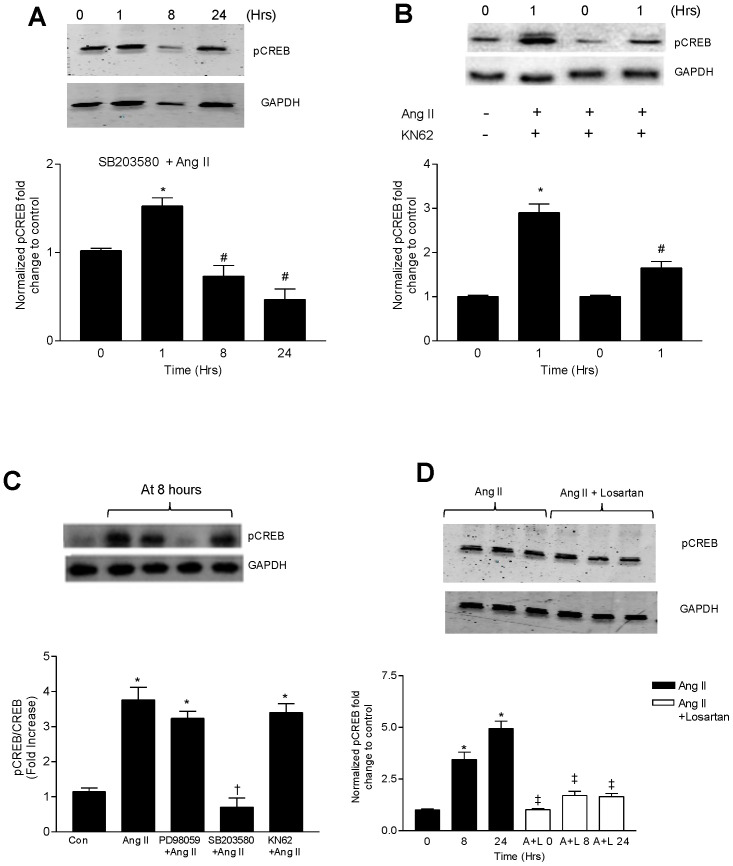
p38MAPK and Ca^++^/CaMK pathways mediate CREB phosphorylation induced by Ang II. Representative immunoblots showing the effect of (A) pretreatment with SB203580 (10 µM) followed by Ang II (100 nM) activation on p-CREB levels over a specified time course. (B) pretreatment with KN62 (10 µM) followed by Ang II (100 nM) at 1 hour on the phosphorylation of CREB. (C) CATH.a cells were preincubated for 30 minutes with PD98059 (25 µM), SB203580 (20 µM) and KN62 (10 µM) followed by Ang II (100 nM) for 8 hours. Cell lysates were immunoblotted for p-CREB. (D) Cells were preincubated with or without Losartan (1 µM) followed by Ang II (100 nM) for 8 hours. At the end of the specified time period, cell lysates were tested for p-CREB levels by western blot. *p<0.05 vs control, ^#^p<0.05 vs 1 hr Ang II and ^†^p<0.05 vs corresponding Ang II group ^‡^p<0.05 vs 8 and 24 hr Ang II treatment. Values are expressed as mean ± SEM. n = 3–4.

Phosphorylation of CREB can be modified through the activation of numerous kinase dependent pathways including CAMKs and p38 MAPK. We therefore examined if either of these pathways are responsible for Ang II-induced CREB phosphorylation (p-CREB). Thirty minutes prior to Ang II stimulation, pretreatment of CATH.a cells with SB203580 (10 µM), a p38MAPK inhibitor, significantly reduced p-CREB levels at 8 and 24 hour time points (Fig. 2A). However, there appeared to be no inhibitory effect on p-CREB levels at 1 hour. 10 µM KN-62, a CAMK inhibitor, significantly blocked phosphorylation of CREB at 1 hour (Fig. 2B) but did not prevent CREB phosphorylation at 8 hours (Fig. 2C). Taken together, these results indicate that early activation of CREB likely occurs through the CAMK mediated pathway but with prolonged Ang II stimulation, the p38MAPK is the predominant pathway. PD98059 (10 µM), an ERK inhibitor, did not significantly alter p-CREB levels at 8 hrs of Ang II stimulation (Fig. 2C). Given that these kinase pathways are all activated by Ang II stimulation, we next tested if blockade of AT_1_R with 1 µM losartan inhibited the Ang II-mediated increase in p-CREB. Losartan completely prevented the Ang II-induced CREB phosphorylation at all time points (Fig. 2D).

### The Effects of CREB knockdown on Downstream Activation of NF-κB, Elk-1, c-Fos and AT_1_R

**Figure 3:**
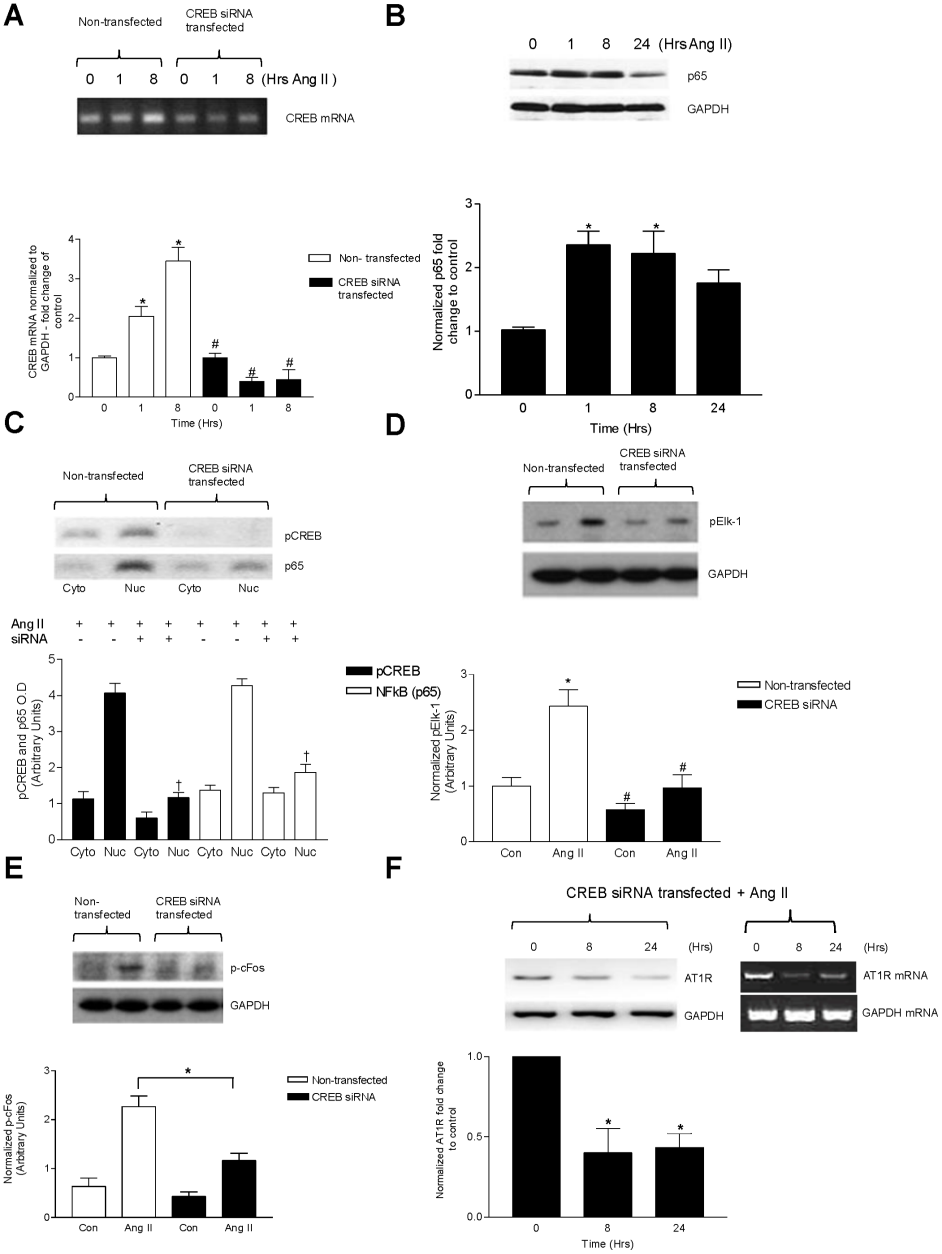
CREB contributes to Ang II-mediated increases in AT_1_R, NF-κB, active Elk-1, c-fos. Knockdown of CREB by siRNA in CATH.a cells. (A) RT-PCR and Western blotting (lower panel) showing efficient suppression of CREB mRNA in CATH.a cells. (B) Inhibitory effect of CREB siRNA on Ang II-induced p65 NF-κB activation. siRNA transfected cells were stimulated with Ang II (100 nM) for 1 hour. p65 NF-κB levels were detected by western blotting. (C) Effect of CREB siRNA on p65 NF-κB nuclear translocation. Cells were transfected with CREB siRNA and subjected to Ang II activation over 1 hour period. Following nuclear/cytoplasmic fractionation, the lysates were immunoblotted for p-CREB and p65 NF-κB levels by western blotting in both nuclear and cytoplasmic fractions. (D) CATH.a cells were transfected with anti-CREB siRNA followed by Ang II stimulation for 8 hours. The cells were then probed with specific antibody against pElk-1 and (E) p-cFos. (F) Cells were either mock transfected with vehicle or anti-CREB siRNA and subsequently stimulated with Ang II (100 nM). Following the specified time course, the cells were probed for AT_1_R message (lower panel) and protein levels. Values are expressed as mean ± SEM. *p<0.05 vs control, #p<0.05 vs Ang II, †p<0.05 vs corresponding untreated nuclear fraction. n = 3–4 per treatment.

We next assessed the contributions of CREB to the activation of NF-κB, Elk-1, c-Fos and upregulation of AT_1_R by silencing CREB with siRNA. To first confirm that the siRNA effectively decreased CREB, we assessed both mRNA and protein expression following siRNA transfection (Figure 3A). There was a significant decrease in CREB mRNA and protein detected in siRNA treated cells which persisted even following 1 and 8 hr Ang II stimulation. We then examined the effects of silencing CREB on p65 NF-κB expression following 1, 8, and 24 hr Ang II treatments (Fig. 3B). Ang II-mediated increase in total NF-κB protein persisted despite CREB silencing at all time points of Ang II stimulation. In order to act as a transcription factor, NF-κB must translocate from the cytosol into the nucleus [25]. Interestingly, the absence of CREB led to a marked reduction in Ang II-mediated translocation of NF-κB in the nuclear fraction (Fig. 3C), suggesting that although total NF-κB protein was unchanged, CREB may play a role in the activation and subsequent nuclear translocation of NF-κB.

Because Elk-1 and AP-1 have previously been reported to be involved in AT_1_R upregulation we next examined the effect(s) of silencing CREB on activation of these transcription factors and the subsequent upregulation of AT_1_R following Ang II stimulation [7], [8]. Silencing CREB led to a decreased Ang II-mediated phosphorylation of both Elk-1 (Fig. 3D) and cFos (Fig. 3E). CREB gene silencing also prevented the Ang II-mediated increase in AT_1_R protein formation (Fig. 3F).

### Luciferase-reporter Assay as Indices of NF-κB and CREB Pathway Activity

**Figure 4:**
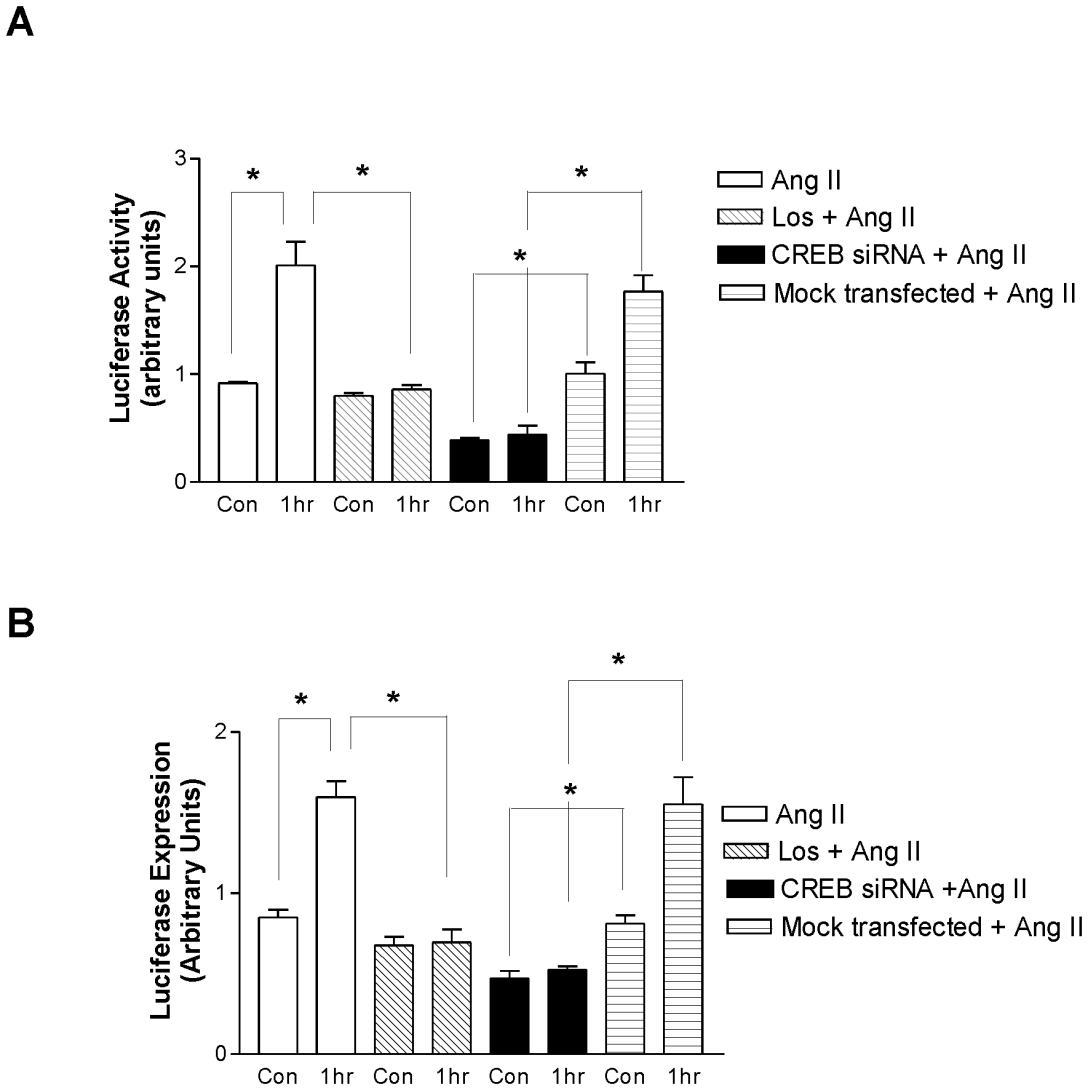
Ang II increases CREB and NF-κB activity in an AT1R and CREB-dependent manner. CATH.a neurons were transfected with CREB and NF-κB reporter constructs, consisting of a mixture of CREB and NF-κB - responsive luciferase constructs and a constitutively expressing Renilla construct. (A) NF-κB relative luciferase activity was quantified under several experimental conditions with Ang II treatment alone or pretreatment with losartan, anti-CREB siRNA or mock transfected cells. (B) CREB luciferase activity was measured under the similar experimental conditions. Values are expressed as mean ± SEM. *p<0.05. n = 3.

Because Ang II increases CREB thereby stimulating NF-κB, we next investigated the interaction of CREB and NF-κB directly using CREB and NF-κB luciferase-reporter assays. As expected, we saw a substantial increase in NF-κB luciferase expression following Ang II which was abrogated by pretreatment with Losartan (Fig. 4A). Cells that were transfected with CREB siRNA alone had decreased NF-κB luciferase activity compared to mock transfected non-stimulated cells, suggesting that CREB may drive basal NF-κB activation. CREB siRNA transfected cells that were stimulated with Ang II did not undergo any increase in NF-κB luciferase activity above CREB siRNA control. Further, this activity was significantly lower than mock transfected cells (Fig. 4A). CREB luciferase expression was also increased following Ang II treatment in an AT1R- dependent manner, as this was blocked by losartan pretreatment (Fig. 4B). As expected, silencing CREB with siRNA similarly caused a marked reduction in CREB luciferase activity following Ang II stimulation as compared to mock transfected control cells (Fig. 4B).

### Effect of Angiotensin II on the Interaction of CREB and NF-κB-DNA Binding

**Figure 5:**
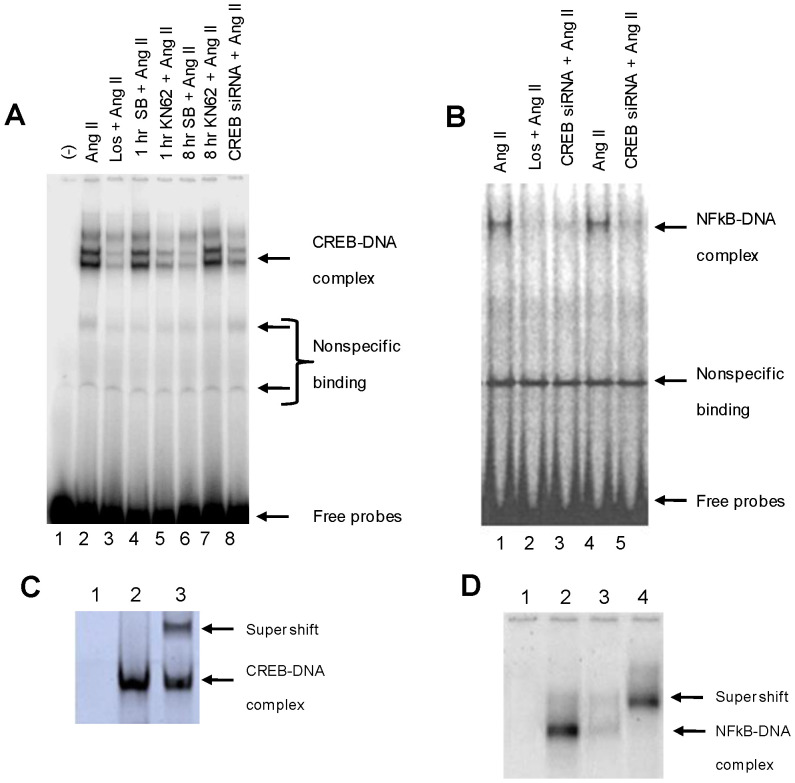
Ang II stimulates CREB and NF-κB binding to DNA. (A) CREB-DNA binding activity was determined under different treatment conditions using a CREB specific labeled probe. (Lane 1) No nuclear extract (negative control). (Lane 2) Ang II (100 nM). (Lane 3) Losartan (1 µM)+ Ang II (100 nM). (Lane 4) SB203580 (20 µM) + Ang II (100 nM) at 1 hour. (Lane 5) KN62 (10 µM)+ Ang II (100 nM). (Lane 6) SB203580 (20 µM) + Ang II (100 nM) at 8 hour. (Lane 7) Non-transfected cells (Ang II (100 nM). (Lane 8) Transfected cells (CREB siRNA)+ Ang II (100 nM). (**B**) To study the synergistic actions of CREB and NF-κB, we conducted EMSA reactions, using NF-κB specific labeled probes under different conditions. (Lane 1) Ang II (100 nM). (Lane 2) Losartan (1 µM)+ Ang II (100 nM). (Lane 3) Transfected cells (CREB siRNA)+ Ang II (100 nM). (Lane 4) Ang II (100 nM). (Lane 5) Transfected cells (CREB siRNA)+ Ang II (100 nM). (C) To confirm the specificity of the CREB-DNA binding bands we did competitive (Lane 1) and supershift assay (Lane 3) using anti CREB antibody, compared to normal CREB-DNA binding (Lane 2). (D) To confirm specificity of the NF-κB -DNA bands, we similarly performed competitive assay (Lane 3) and supershift assay using anti-p65 antibody (Lane 4). Normal binding and negative control using no nuclear lysate are also included (Lane 2 and Lane 1, respectively). Fig. 5C is excluded from this article's CC-BY license. See the accompanying Expression of Concern for more information.

In order to determine the transcriptional activity at the DNA level, we performed EMSA on CATH.a cells treated under different experimental conditions using consensus probes for both CREB and NF-κB. As a negative control, a sample containing no nuclear extract was used to confirm specificity (Fig. 5A, lane 1). Ang II stimulation of the CATH.a cells for 1 hour resulted in the formation of two prominent protein-DNA complexes (Fig. 5A, lane 2), which was prevented by 30 minute pretreatment with losartan or KN62 prior to Ang II stimulation (Fig. 5A, lane 3 and lane 5). Treating the cells with SB203580 prior to Ang II stimulation had no short term effect on the DNA binding at 1 hour, but DNA-protein binding was reduced at the 8 hour time point (Fig. 5A, lane 4 and lane 6). siRNA mediated CREB silencing resulted in a significant decrease in the DNA-protein binding (Fig. 5A, lane 8) as compared to the non-transfected control (Fig. 5A, lane 7).

We then examined the ability of CREB to influence the protein-DNA binding function of NF-κB using NF-κB-specific probes for EMSA reactions. Ang II caused the formation of a prominent DNA-protein complex (Fig. 5B, lane 1) which was prevented by pre-treatment with losartan (Fig. 5B, lane 2). Treatment of the cells with Ang II following CREB siRNA mediated gene silencing decreased DNA binding to NF-κB (Fig. 5B, lane 3) as compared to the non-transfected cells (Fig. 5B, lane 4). These data are consistent with our western blot analyses which showed a decrease in NF-κB nuclear translocation as a result of CREB inhibition. In this set of experiments, we again ran a negative control without nuclear extracts (Fig. 5B, lane 5). Taken together, the EMSA data corroborate the western blot analyses. The specificity of the NF-κB and CREB–DNA binding was confirmed by supershift assays using anti-p65 NF-κB and anti-CREB antibodies as well as performing competitive binding assays in the presence of excess cold probes (Fig. 5C and 5D).

### NF-κB and CREB Interaction is Facilitated by CBP

**Figure 6:**
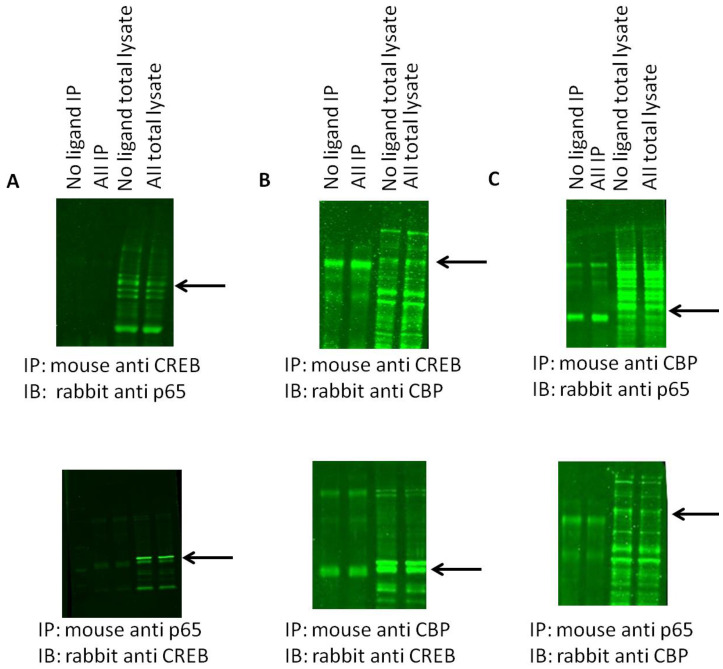
CREB and p65 NF-κB do not physically associate and CBP binds to both CREB and p65 NF-κB. All representative blots are in the following order: Lanes 1–4: immunoprecipitated cell lysate, no ligand, Ang II, Los, Ang II+Los; Lanes 5–8: Total lysate, no ligand, Ang II, Los, Ang II+Los. (**A**). CREB (C) and p65 NF-κB do not associate. As shown in the first four co-IP lanes, there is no detectable signal but total p65 NF-κB was still detected. (**B**). CBP and p65 NF-κB physically associate as shown in the first four lanes. (**C**). CBP and CREB physically associate.

We next asked if there was both a physical and functional interplay between CBP, CREB, and NF-κB. Co-immunoprecipitation (co-IP) analyses indicated there was no physical association between CREB and NF-κB (Fig. 6A, top and bottom panels). However there is physical binding between CBP and NF-κB (Fig. 6B) as well as CBP and CREB (Fig. 6C). To validate the co-IP experiments, we also repeated the experiments in the presence of blocking peptides against CBP or CREB (Figure S1). Indeed, in the presence of blocking peptide against either CBP or CREB, no positive bands were detected in either co-IP or total lysate. Our results therefore show that CBP acts as a facilitator to the binding of both NF-κB and CREB to their respective DNA domains independent of one another.

## Discussion

In the present study we have demonstrated that Ang II stimulation leading to AT_1_R upregulation is mediated by an orchestrated sequential activation of the transcription factors CREB, NF-κB, Elk-1 and cFos. Our results are in agreement with previous reports that Ang II via AT_1_R indirectly activates CREB by phosphorylation of Ser133 [18]. A new finding in this study is that CREB acts coordinately with other transcription factors in regulating AT_1_R expression. We have further elucidated the key pathways resulting in CREB activation by Ang II, namely early activation by the CAMK pathway followed by sustained activation by the p38MAPK pathway. Finally, we have shown that CREB activation is critical for the nuclear translocation of the p65 component of NF-κB, which triggers the cascade of transcription factor activity leading to AT_1_R upregulation in response to Ang II.

Angiotensin II is the central member of the RAS. Overactivity of the RAS is a hallmark of several disease states characterized by sympathoexcitation such as heart failure and hypertension. Increased circulating Ang II and an upregulation of the AT_1_R are common manifestations in sympathoexcitatory states [2], [3], [26]. AT_1_R protein is increased in the sympathoexcitatory regions of the brain such as the RVLM. This occurs as a result of a positive feedback mechanism which can be blocked by the AT_1_R blocker losartan.

We have previously shown that AT1R upregulation is dependent on the increased activation of NF-κB [7] and AP-1 [8], [27] and simultaneous activation of Elk-1 [7]. Since NF-κB does not bind to the promoter site of cFos, Elk-1 may serve as the bridging protein thereby indirectly influencing cFos transcription [28]. Increasing evidence suggests that transcription factors work in a regulated network with one another to affect end point cell signaling [29], [30], [31]. The possible reasons for this co-dependent mechanism may be (a) to exert greater control over the end protein transcription or (b) redundant pathways regulating short term or long term effects of cell signaling initiators such as Ang II.

CREB is activated in neuronal cells and is involved in the transcription of genes which are involved in varied neurological processes ranging from behavioral adaptation [32] and learning [33] to targeting other transcription factors including c-Fos [13], which is intimately involved in AT_1_R gene transcription. Ang II activates several signaling pathways leading to the phosphorylation and subsequent activation of CREB at Ser-133. From our results, we have found that there are two distinct pathways which phosphorylate CREB; (a) early phosphorylation by Ca^2+^/calmodulin-dependent protein kinase II which can be blocked by KN-62 and (b) a late phosphorylation process mediated by p38 MAPK that can be blocked by SB203580. Ang II has previously been known to activate p38 MAPK [34] most likely by the mitogen and stress activated protein kinase (MSK-1) [35]. Intracellular calcium and activation of the Ca^2+^/CAMK pathway is also crucial for the Ang II mediated CREB activation [36]. Previous studies have shown that CAMK can phosphorylate CREB [13]. Our results clearly demonstrate that CAMK is involved in the early phosphorylation of CREB. Further evidence that CREB activation occurs through the Ang II-AT_1_R axis is demonstrated by the fact that this process is blocked by losartan, a specific AT_1_R blocker.

The indirect interaction between CREB and NF-κB may be a method of cross-talk between NF-κB and CREB signaling pathways to compliment the integration of their signaling pathways together with their affinity for acting as a bridge to bring these molecules to the target promoter sites, since CREB and p65 NF-κB interact with different regions of the coactivator. Co-operation between the two molecules exists as the CREB promoter contains binding sites for CREB in close proximity to p65 motifs [21]. NF-κB has been shown to regulate the activity of other bZIP family members related to CREB (ATF-2, c-Jun, and c-Fos) [22], [37]. The data presented here show that when CREB is silenced by siRNA, basal NF-κB activity is decreased.

NF-κB and c-Fos (as part of AP-1) have been implicated as key molecules in the pathophysiology of heart failure and hypertension; despite being activated via different pathways, they share similar activation stimuli [38]. Both of these proteins have also been implicated in AT_1_R upregulation *in vitro* [7].

**Figure 7:**
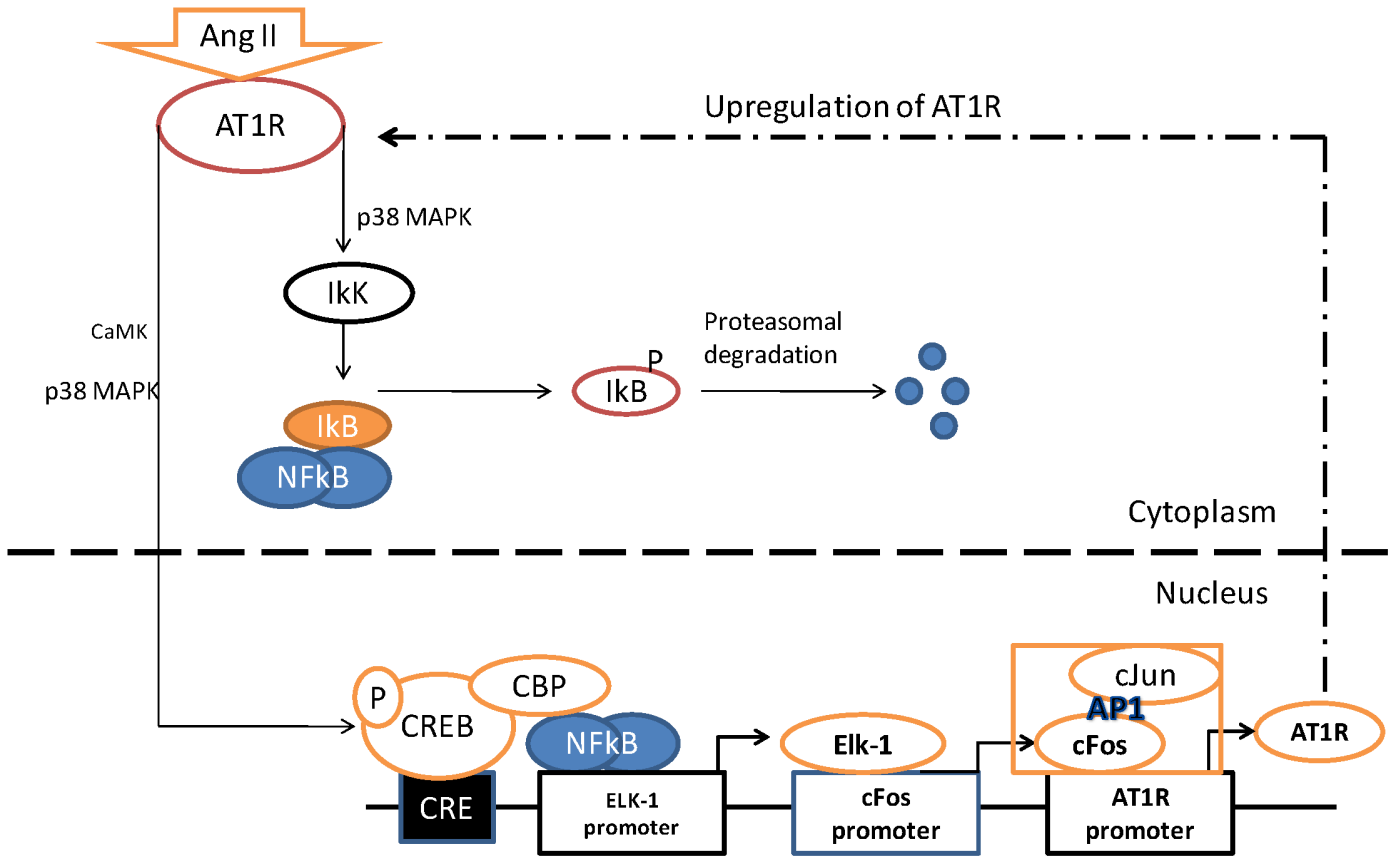
A proposed model of Ang II-mediated AT_1_R upregulation. In response to an Ang II stimulus, NF-κB disengages from IkB and enters the nucleus to interact with the CBP and binds to the promoter region of Elk-1. In addition, CREB and CBP complex formation facilitates the interaction between p65 and the Elk-1 promoter. This sets up a downstream cascade mechanism involving the increased transcription of Elk-1 and the the c-Fos component of AP-1 eventually leading to the upregulation of AT1 receptor.

In the present study, we have demonstrated that in neuronal cells CREB-NF-κB interaction via CBP is necessary for downstream activation of other transcription factors including Elk-1 and AP-1 leading to the upregulation of the AT_1_R as a result of Ang II activation. A summary of the pathway that our data support is outlined in Fig. 7.

### Limitations

The goal of these experiments was to study the Ang II – AT_1_R axis in neuronal cells. We accept that our cell model, the CATH.a neuron may not exactly mimic the cellular or molecular mechanisms or exhibit the identical phenotype of an adult neuron found in sympathetic regulatory regions of the brain. An alternative is to use primary cultures from neonatal or adult brains of *in vivo* models. But these models too have their inherent limitations. Neonatal cells may not react in a similar way to adult neuronal cells and hippocampal cell cultures may not reflect the molecular or signaling events occurring in neurons from the brain stem. The CATH.a cell line, despite its limitations, has been used for study of AT_1_R, JNK [8], NF-κB [7], Elk-1 [39] and AP-1 [40] pathways. CATH.a cells have also been used for numerous studies involving CREB activation and mechanisms [41], [42], [43]. Therefore, we believe that this cell line is adequately close to and has been validated as a viable model for studying transcription factor activities and signaling pathways. Ultimately, these data will have to be validated in an animal model of sympathoexcitation.

In summary, we have for the first time demonstrated that CREB activation is necessary for AT_1_R protein synthesis in response to Ang II stimulation in neurons. Silencing the CREB gene using siRNA results in abrogation of the transcription factor activation of NF-κB, Elk-1 and cFos, resulting in decreased synthesis of AT_1_R protein. Furthermore we have shown that CBP acts as a transcriptional co-activator and integrates the binding of p65 and CREB to their respective promoter sites. Our results will further aid in understanding the mechanism of Ang II induced neural sympathoexcitaion as seen in many disease states.

## Supporting Information

Figure S1 **Blocking peptides against CREB or CBP prevent all antibody binding.** All representative blots are in the following order: No ligand IP, Ang II IP, no ligand total lysate, Ang II total lysate. (A) Pre-incubation with a blocking peptide for the rabbit anti-CREB antibody prevents any detection of either CBP/CREB dimeric species or CREB protein in total lysate. (B) Pre-incubation with the blocking peptide for the rabbit anti-CBP antibody prevents detection of either CREB/CBP dimeric species or CBP protein in total lysate. (C) Pre-incubation with a blocking peptide for the rabbit anti-CREB antibody prevents detection of any potential CREB/p65 NF-kB dimeric species and CREB total protein. (D) Pre-incubation with the blocking peptide against the rabbit anti-CBP antibody prevents the detection of both p65 NF-kB/CBP dimeric species and CBP total protein.
(DOCX)
