# Expression of Concern: NF-κB and CREB Are Required for Angiotensin II Type 1 Receptor Upregulation in Neurons

**DOI:** 10.1371/journal.pone.0294092

**Published:** 2023-11-02

**Authors:** The PLOS ONE Editors

Following the publication of this article [1], it was noted that Fig. 5C appears to partially overlap with Fig. 5B of an article with no shared authors [2] that was published at an earlier date.

The corresponding author stated that they were unable to locate the original underlying data for this figure, and they acknowledged that the image in this article [1] appears to have been taken from the previous article [2].

While original data underlying some of the other figures in this article was available, the corresponding author acknowledged that much of the original data is no longer available due to the age of the article.

The *PLOS ONE* Editors issue this Expression of Concern to notify readers of the issue in Fig. 5, which raises concerns regarding the reliability of the data presented in this figure.

Fig. 5C appears to report material from [2], published in 2010 by The Company of Biologists, which is not offered under a CC-BY license and is therefore excluded from this article’s [1] license. At the time of publication of this Expression of Concern, the original article [1] was republished to note this exclusion in the Fig. 5C legend and the article’s copyright statement.
