# Supplementary material for: NF-κB and CREB Are Required for Angiotensin II Type 1 Receptor Upregulation in Neurons
Source: PLoS One. 2013 Nov 11;8(11):e78695. doi: 10.1371/journal.pone.0078695 (PMC3823855; doi:10.1371/journal.pone.0078695)
Supplement: Figure S1 — Blocking peptides against CREB or CBP prevent all antibody binding. All representative blots are in the following order: No ligand IP, Ang II IP, no ligand total lysate, Ang II total lysate. (A) Pre-incubation with a blocking peptide for the rabbit anti-CREB antibody prevents any detection of either CBP/CREB dimeric species or CREB protein in total lysate. (B) Pre-incubation with the blocking peptide for the rabbit anti-CBP antibody prevents detection of either CREB/CBP dimeric species or CBP protein in total lysate. (C) Pre-incubation with a blocking peptide for the rabbit anti-CREB antibody prevents detection of any potential CREB/p65 NF-kB dimeric species and CREB total protein. (D) Pre-incubation with the blocking peptide against the rabbit anti-CBP antibody prevents the detection of both p65 NF-kB/CBP dimeric species and CBP total protein. (DOCX) [file pone.0078695.s001.docx]

**NF-κB and CREB Are Required for Angiotensin II type 1 Receptor Upregulation in Neurons**

**ONLINE SUPPLEMENT**

**Karla K. V. Haack, Amit K. Mitra, and Irving H. Zucker**

Department of Cellular and Integrative Physiology, University of Nebraska Medical Center, Omaha, Nebraska, USA

**Figure S1.** Blocking peptides against CREB or CBP prevent all antibody binding.
